# Characterization of the complete chloroplast genome of *Davidia involucrata* Baill. (Nysssaceae), an endangered species endemic to China

**DOI:** 10.1080/23802359.2019.1699468

**Published:** 2019-12-12

**Authors:** Zhuo Qi, Zhenzhong Jiang, Peng Jiao, Jing Qu, Siyan Liu, Dan Yao, Piwu Wang, Shuyan Guan, Yiyong Ma

**Affiliations:** College of Life Sciences, Jilin Agricultural University, Changchun, China

**Keywords:** *Davidia involucrata*, Baill, endangered species, chloroplast genome

## Abstract

*Davidia involucrata* Baill. is a kind of tertiary paleotropical plant floristic relic species unique to China. This rare plant is disappearing due to poor adaptability and serious poaching. We first assembled the complete chloroplast (cp) genome of *Davidia involucrata* Baill. by Illumina paired-end reads data. The whole genome was 169,085 bp, consisting of a pair of inverted repeats of 169,379 bp, large single copy region and a small single copy region (96,712 and 67,667 bp in length, respectively). The cp genome contained 90 genes, including 64 protein-coding genes, 22 trRNA genes and 4 rRNA genes. The overall GC content of the whole genome was 38.04%. A neighbor-joining phylogenetic analysis demonstrated a close relationship between *Davidia involucrata* Baill. and *Nyssa yunnanensis.*

*Davidia involucrata* is a kind of tertiary paleotropical plant floristic relic species unique to China. *Davidia involucrata* is also named as Chinese dove tree, because the lower bud looks like a white dove ready to fly and has a high ornamental and research value (Shimozu et al. 2017). *Davidia involucrata* belongs to the family Nyssaceae. The plant, which was widely distributed during the tertiary and cretaceous periods, is now reserved only in Sichuan, Chongqing, Hubei, Guizhou, Hunan, Yunnan and Gansu provinces of China (Tang et al. 2017). The dove tree is one of the key protected plants in China. But, it is still seriously threatened by human activities and overexploitation. The living fossil of this plant is gradually decreasing due to poor adaptability and serious poaching.

In this study, *Davidia involucrata* was sampled from the National Nature Reserve of Badagongshan Zhangjiajie County, China (109°41′45ʺE, 29°39′18ʺN). A voucher specimen (JN20195052) was deposited in the Herbarium of the Plant Biotechnology Center of Jilin Agricultural University, Changchun, China.

The present study is the first to assemble and characterize the complete chloroplast genome for *Davidia involucrata* Baill. (GenBank: NC_031376.1) from high-throughput sequencing data. The existing chloroplast Genome sequence of ginkgo biloba was downloaded from the National Center for Biotechnology Information’s Organelle Genome Resources database (nc_031376.1) as the reference sequence, and the chloroplast Genome of ginkgo biloba was assembled using SPAdes v3.6.0 software (Bankevich et al. 2012). The default setting of parameters was adopted. Sequence annotation first confirmed the availability and boundary of genes by BLASTN comparison directly through the protein-coding sequence of the proximal species. Then, the genes in the chloroplast genome were annotated by online tool DOGMA (http://dogma.ccbb.utexas.edu/) with default parameters, and the genes were functionally annotated by combining with NR (http://www.ncbi.nlm.nih.gov/) database (Lohse et al. 2013). TRNA was annotated using the trnascan-se online site. RNAmmer 1.2 Server (HTTP//www.cbs.dtu.dk/services/RNAmmer/) was used to record rRNA for comments. We used Homblocks (Bi et al. 2018) and MEGA6 (Tamura et al. 2013) to reconstruct a neighbor-joining (NJ) phylogeny *of Davidia involucrate* with 1000 bootstraps.

The complete cp-DNA of *Davidia involucrata* Baill. was a circular molecule with 19,085 bp length, comprising a large single-copy (LSC) region of 96,712 bp and a small single-copy(SSC) region of 67,667 bp, separated by two inverted repeat regions (IRs) of 2353 bp. It contained 90 genes, including 64 protein-coding genes, 4 ribosomal RNA genes, and 22 tRNA genes. The phylogenetic tree reveals that all the species of Nyssaceae formed a monophyletic clade with high-resolution value in cornales and *Nyssa yunnanensis* W.C.Yin is closely related with *Davidia involucrata* Baill. (Figure 1).

**Figure. 1.**
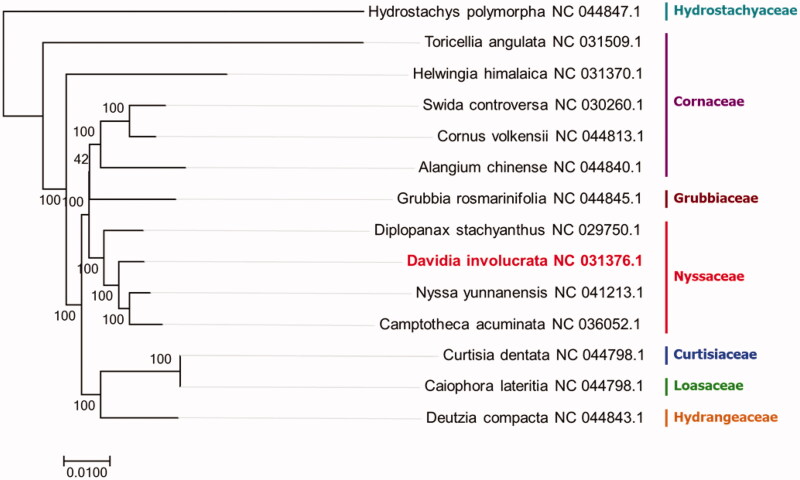
The phylogenetic tree based on 14 cornales complete plastid genome sequences.
